# Goal conditioning throw mutimodal categorisation in a simulation of rat navigation

**DOI:** 10.1186/1471-2202-14-S1-P137

**Published:** 2013-07-08

**Authors:** Souheïl Hanoune, Mathias Quoy, Philippe Gaussier

**Affiliations:** 1 University of Cergy Pontoise, France; 2 ETIS Laboratory, UMR 8051, ENSEA - University of Cergy-Pontoise, CNRS, France

## 

Navigation tasks are based on two approaches: place-action directed or goal directed. In the goal directed ones, a reward is generally given to the system when the goal is reached. Many models are able to predict the reward in a simple cases. In this paper, we present an architecture for complex conditioning.

The purpose is to present a case where an association is used to predict the reward in the task. In this task, the goal is multimodal, i.e. the presence of one of the components is no longer sufficient. To correctly predict the reward, the correlation between two informations has to be saved. The experiment presents how the system can resolve ambiguities.

The hypothesis in our model is that when a hippocampal conditioning is failing, the prefrontal cortex is neuro-modulated to facilitates the categorization of multimodal contexts. The goal is to encode the correlations between the inputs. When the contexts are activated in the future, they will help the conditioning in the hippocampus. The experimental setup is derived from the continuous place navigation task [1,2], where a rat has to find a goal marked by a blue spot, then wait 0.3 s in this location in order to obtain the reward (food). An automated pellet giver producing a sound releases the food. After some time, the sound is linked to the food reward. In the experiment an ambiguity subsists between two locations: two blue spots are present in the environment but only one of them has the sound occurrence. The prediction of the reward is correlated with the correct prediction of the sound, in the sequence place-cell -> blue spot → sound -> food. In this work, we study how failure in the conditioning can be solved by introducing multimodal contexts.

**Figure 1:**
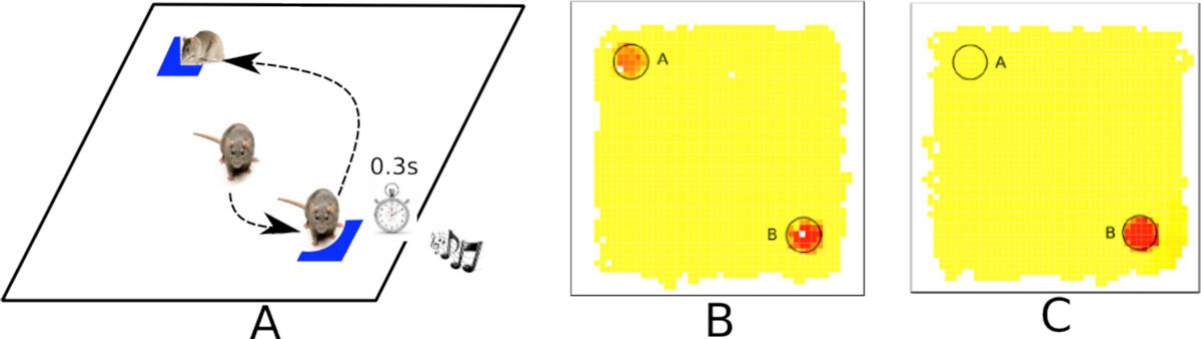
**Panel A presents the experiment**. There are two blue spots in the environment, but the reward is obtained by waiting 0.3s in only one of them. Panel B presents the reward predictions of the rat at the beginning: both of the spots are predicted as a reward. In panel C that the reward is fully encoded in the place B and A is no longer predicted as a reward.
